# On the Management of the Poor in Scotland

**Published:** 1841-01

**Authors:** 


### BRITISH AND FOREIGN

### MEDICAL REVIEW,

FOR JANUARY, 1841.

### PART FIRST.

### Analytical and Critical Reviews.

### Art. I.

1. *On the Management of the Poor in Scotland.* By William Pulteney Alison, m.d., f.r.s.e. Second Edition.—*Edinburgh*, 1840. 8vo, pp. 123.

2. *Report on the Sanatory State of the Labouring Classes, as affected chiefly by the Situation and Construction of their Dwellings in and about the Metropolis.—London*, 1840. 8vo, pp. 30.

3. *Papers relating to the Noxious Effects of Fetid Irrigations around the City of Edinburgh.—Edinburgh*, 1839. 8vo, pp. 94.

4. *An Examination of the Statements contained in the Papers relating to the Fetid Irrigations around the City of Edinburgh.* By William Tait, Surgeon.—*Edinburgh*, 1839. 8vo, pp. 40.

5. *Vital Statistics of Glasgow.* By Robert Cowan, m.d.—*Glasgow*, 1838. 8vo, pp. 54.

6. *Hygiène Publique, ou Mémoires sur les Questions les plus importantes de l'Hygiène, appliquée aux professions et aux travaux d'utilité publique.* Par A. J. B. Parent-Duchatelet.—*Paris*, 1836. Deux Tomes. 8vo, pp. 552, 708.

*Public Hygiène, or Memoirs on the most Important Questions of Hygiène, applied to professions and to works of public utility.* By A. J. B. Parent-Duchatelet.—*Paris*, 1836. Two Vols.

Notwithstanding the confident manner in which many very respectable authorities, both new and old, have expressed themselves respecting the Causes and Propagation of Continued Fever, we believe there never was a time when the opinions of reflecting men were more generally suspended or less decided regarding them than at present. For this reason, although we are aware that enquiries are now on foot which promise to adduce much additional information from all parts of the country, we do not think it premature to examine and weigh the materials already in our possession, and to state the results which may be legitimately deduced from them. If we were to content ourselves with mere opinions, the difficult subjects under discussion would admit of being summarily dismissed in one or other of two ways,—either by selecting from the mass those opinions which are sanctioned by the most distinguished authors, or by receiving without investigation—as seems to have been the custom—every notion that has been entertained regarding them. But we must not forget that the present may be styled as much the age of discrimination and independence as of discovery and enterprise. Men are not now satisfied with knowing an *opinion;* they must take to pieces the machinery by which it is elaborated, and inspect the materials from which it is made, before they decide upon its merits. We see every day evidences of the searching spirit of our times in the crumbling fragments of long-cherished hypotheses which now only litter the path of science; and we would hope that this genius of a better philosophy will soon add to its other triumphs in medicine substantial and intelligible doctrines on the matters in question, fit and worthy to supplant the crude and misty conceits which have so long passed current in their stead.

While it must be allowed that fevers have received their full share of accommodation in the libraries of medicine, in these as in former times, it cannot be with equal justice maintained that the voluminous records of their history have been generally composed in the exercise of a sound and discriminating judgment. Numerous, if not imperishable, are the monuments of industrious contention which have been reared in almost every European language on the track of epidemics. Their origin, diffusion, nature, treatment, have each been the ground of dispute, and still there is but little that can be considered established; men continue to think apart and to think differently. This state of matters we hardly believe to be altogether warranted by the condition of our knowledge of fever, imperfect as it confessedly is. We conceive that enough is ascertained to convince an unbiassed enquirer that our common continued or contagious fever is a disease diversified, not in *kind* but in its degree and external features only, according to the many modifying circumstances which affect it in different classes, ages, situations, and seasons; whether, as Dr. Haygarth remarks, it be "denominated the slow, low, nervous, putrid, petechial, malignant, pestilential, jail, ship, camp, hospital fever," synochus, or typhus. We believe also that enough is ascertained to prove that it is not a local disease, not an inflammation of the brain, of the bronchi, of the blood-vessels, or of the intestines; and that, moreover, there are materials in existence adequate to instruct us—if not indisputably on what the origination of fever depends, at least on what it does *not* depend—a kind of information which, though not the precise thing we most desire to know, is yet of much value in narrowing the question, and which, when fully matured, will shut up the enquiry within very small limits, if it do not enable us to ascertain positively the true causes of fever with certainty.

It would be an idle task to endeavour to ascertain the source of fever while we attach no precise notions to the disease itself, and while we remain in doubt whether it is a specific disease originating under peculiar circumstances, or a disorder of an indeterminate nature, capable of being excited indifferently by a multiplicity of external and dissimilar agents.

While certain specific diseases, such as smallpox, are conceived always to be produced by specific poisons or contagions, it has been a very common supposition, even with those who admit that fever is disseminated by contagion, that its origination may be the effect of external matters by no means fixed or definite in their nature, of a common or general infection, as they term it, by way of distinction from that which is fixed and peculiar. In so far as this doctrine maintains that noxious effluvia of different kinds may produce febrile disorders, of a continued and even typhoid kind, there can be no reasonable doubt of its truth; yet he who admits that the manifold emanations from putrefying carcasses and decaying vegetation, the corrupted exhalations of overcrowded apartments, the steams of the many obscene mixtures which pollute the hovels of the poor, and such other malarial productions as have enjoyed the evil reputation of generating typhus, are capable of producing a fever—may believe that they have no more power to produce our common *contagious* fever than they have to produce measles or smallpox, which may frequent the same localities, and yet cannot be referred to a malarial cause unsettled in its origin and indeterminate in its constitution. It may indeed be maintained that the analogy of certain fevers of definite characters and career, originating under very different conditions, gives support to the supposition that our common fever may be produced by different agents. The yellow fever at one time rages in the low marshes of Fort Augusta, at another on the dry elevation of Stoney Hill; agues abound in the fens of Holland and in the arid and elevated province of Quito, as at Guaillabamba and Salinas de Mira; but it does not follow that the essential causes of these fevers are different, merely because the external features, and more familiar qualities of soil and atmosphere are unlike in those places. The argument that might be founded on the dissimilarity of the local conditions in either case, as tending to prove a dissimilarity in the causes of those fevers, has far less weight than the opposite argument has—of the similarity of the real, though unknown causes being implied by the sameness of the effects. We shall by and bye endeavour to ascertain what grounds there are for supposing, not simply that contagious fever is capable of being produced by several products of a malarial kind, but that it can be produced by any malarial substance whatsoever.

While, on the one hand, the supposed multiplicity of causes from which contagious fever might originate, has tended to prevent the disease from being looked on as of a specific nature, the fact, on the other hand, of typhoid symptoms being, under particular circumstances, common to it with many other disorders, has still farther countenanced the idea of its being devoid of a specific character. There seems also in the minds of some to be a vague notion of the peculiarity of typhus fever, at the same time that it is conceived to be, in certain circumstances, a mere aggravation of other forms of disease. "Typhus fever," says a recent writer, "is not only an endemic disease *sui generis*, but so strong is the predisposition to that form of *pyrexia* that it is prone to become an aggravation and superaddition to other forms of fever; and all the remittent types and degrees, as well as the catarrhal and peripneumonic fevers, are apt, either when long continued or improperly treated under a heating regimen, to glide into it." We apprehend that a good deal of the confusion which exists among us on the subject of fever depends on the importance which has been attached to the *typhoid state.* Nor will this confusion subside until medical men shall learn to consider the typhoid state as merely a group of symptoms which, while it is often ingrafted on common contagious fever as it sometimes is on smallpox, remittent fever, &c., yet is no more an essential part of such contagious fever than of those other diseases. It is surely unnecessary for us to prove that the fever with which the population of this country is so often and so extensively affected, has peculiarities which do not admit of its being confounded with every disorder which may assume a typhoid appearance. Viewed in the mass, and not in single cases and exceptions, it has a definite progress and succession of symptoms, has particular periods of increase and decline, and possesses unequivocally the property of diffusing itself by contagion. When the fevers which have been ascribed to divers malarial substances, and which may have exhibited the typhoid state, shall be found to possess all these peculiarities of the common contagious fever, we shall admit their identity with it; but another question will then remain to be discussed—Have these fevers originated from the causes to which they have been referred?

It is generally admitted that the contagious fever of this country may be and often is generated anew. At the same time there are some who hold with Dr. Bancroft, that this fever is never produced but by the specific contagion, which they presume never to be extinguished; but which having been "the original work of our common Creator, must have been continued in existence by the energies of a living principle, exerted successively in the different bodies through which it has been transmitted from one generation to another." (Bancroft, p. 109.) To reconcile this doctrine with experience the contagion of malignant fevers has been shut up for years, according to some "in holes, and chests, and caves," and has even made its hiding-place in spiders' webs.

As much of the matter in the works before us has been accumulated on the supposition that the continued fever of this country is capable of being generated *de novo*, we shall assume, for the present, the common opinion to be true. Nor does it appear that there need be much hesitation in making the assumption, since it is so much in accordance with the convictions of many practical men of acute discernment. Dr. Bateman says on this subject: "We cannot doubt that a great number of the cases of fever which occur during an epidemic season are entirely independent of contagion for their origin." (p. 12.) Dr. Pritchard, too, observes that "the instances are very numerous in which fever has arisen under circumstances almost precluding the possibility of an origin in contagion; and so many examples of this description have fallen under my own observation, as fully to persuade me that this disease does originate spontaneously or independently of communication with any infected body." (Fever in Bristol, p. 94.) Dr. Percival, and a host of other practical observers, hold the same opinions.

Of the many notions which have been promulgated in respect to the origin of fever, a sufficiently distinctive arrangement may be made under two heads: 1st. That there is a "fever-poison," generated from sources exterior to the living body. 2d. That certain physical and moral conditions may so act on the operations of the body as to cause it to generate within itself that which produces the phenomena of fever, independently of any exterior poison.

We cannot afford space to recount the many sources to which the fever-poison has been referred, nor the many authors who have yielded their testimony in favour of the generation of this substance exterior to the living body. All that is of real importance in these long-cherished opinions will fall to be considered as we proceed in our examination of the works before us. We turn in the first instance to the "Reports of the Sanatory State of the Labouring Classes in and about the metropolis." In the Report by Drs. Arnott and Kay we have the following passage:

"Besides the malaria arising where nature is uncultivated, we find that, wherever men congregate and bring together the quantities of vegetable and animal substances which constitute their food, in the preparation of which there is much refuse, or where the excrementitious matters from their own bodies (being the matter of their food again rejected, and in another form,) are allowed to accumulate, there is produced another malaria, often as destructive to life as the most active which dwells in an Indian jungle. The fevers called typhus, putrid, malignant, jail, hospital, ship-fever, &c., are the produce of this malaria, and, when once induced, the bodies of persons affected give out a contagious malaria, often more quickly operative on other persons than the original cause." (p. 12.)

In confirmation of this doctrine there is adduced a series of communications from district surgeons tending to connect the prevalence of fever with the existence of various sources of the malaria in question. First, there are references made to the "imperfection, or want of sewers and drains." The following is a specimen of the evidence on this point, from the pen of Mr. Wagstaffe, a parochial surgeon in Lambeth:

"There are, at the present time, many cases of severe fever in and about the parish above alluded to, which have continued for some time.......The primary cause of this infection I believe to be the malaria or effluvia arising from the state of the drains or stagnant filth: the heat of the sun acting upon the mud sends forth this kind of malaria, which, impregnating the air, is the first cause of fever." (p. 17.)

Next, "the existence of uncovered and stagnant drains or ditches, containing vegetable matters in a state of decomposition," is referred to, and its connexion with fever maintained in a communication from the district surgeon, Mr. James Appleton. Alluding to Saffron Hill, which is selected as a case in point, the communication proceeds,

"First, there is an open sewer running the whole length of the district, not a drain but almost a river of filth, which passes under Farringdon street and Bridge street (where it was formerly known by the name of the Fleet ditch), and empties itself at the foot of Blackfriars Bridge: upon the very edge of this ditch many of the poor have their dwellings, so that they may be said to live continually in an atmosphere tainted by it. Next, I may mention that some of the privies in the neighbourhood of this sewer are in a very sad condition; and lastly, the great mass of the houses in this neighbourhood are exceedingly dirty, and contain as many inhabitants as they well can. The great majority of the cases of sickness occurring in this district are in the locality above alluded to; and the diseases most prevalent since I have had the charge (six years and a half) are typhus and continued fevers. Six years ago fever prevailed very much in this particular neighbourhood; and again, for nearly the last two years, we have never been quite free from it. Many of these fever cases become chargeable to the parish, in some shape or other, principally by being admitted into the workhouse, in which we have been obliged to appropriate one ward to fever cases, and which has seldom been empty for the last eighteen months. The number of fever cases occurring in the year 1837 was about 200; of these about sixty cases were treated in the workhouse. No doubt some of these cases came from various parts of the district. Considering the filthy habits of the people dwelling in this particular locality; considering the privations many of them undergo with respect to food, and their intemperate use of ardent spirits; that they are huddled together in ill-ventilated rooms, and that this place is the resort of Irish lodgers who are travelling the country, *it is exceedingly difficult to give an opinion how far these cases of fever have had their origin in such causes; how far they have had their origin in states of the atmosphere equally affecting the crowded parts of this metropolis; and how far they have originated in the local causes above named;* especially as I must not omit to mention that during three years we had very few cases of fever, and also that this part was very lightly visited by spasmodic cholera, not more than a hundred cases occurring, so far as I know, from its first appearance in this town." (p. 18.)

Then, thirdly, we are introduced to "open stagnant pools of water, rendered putrid by the admixture of animal and vegetable substances," on the evil influences of which the report of the district surgeon is as follows:

"I may state that in my district, comprising Homerton and Mare street, of the Hackney union, I am seldom without cases of a typhoid character, and have carefully searched through my register of sickness from Lady-day 1837 to Lady-day 1838, and find there have been twenty-four cases of severe typhus, of which four were fatal; fifteen of the number were in one locality, named Silk-Mill-row, and Wick street, attributable, I think, to an obstruction by a dam to a mill, which allows a large accumulation of decaying and other matter of a deleterious nature, likely to cause an atmosphere not at all congenial to health, which, aided by, I am sorry to say, the innate want of cleanliness and care on the part of the poor, frequently gives rise to fevers of this description, notwithstanding my very urgent and strenuous endeavours to inculcate the importance of it to their own welfare and comfort." (p. 19.)

Fourthly, we are told,

"Undrained marsh-land is mentioned as a cause of fever in Great Stanmore parish, Edgeware, and the medical officer, Mr. Foote, urges the draining of the marsh at the public expense. Two years past a fever raged at Red Hill, which I attributed to the lodgment of the filth from privies, which I had removed at the time; and the same thing occurred at the Hyde, the fever prevailing there being of the typhoid type; and I consider that unless the ditch is cleaned the same kind of fever will prevail again; and also at the Marsh, in the parish of Great Stanmore, typhus fever lately prevailed amongst the poor." (p. 19.)

And this suffices to *prove* the influence of marsh-land!

Fifthly, "accumulations of refuse, either thrown from the houses or otherwise collected in the streets, courts, and lanes," are specified as causes of fever, and the principal communication on these is Mr. Bowling's letter of May, 1838.

"In reply to your letter of the 27th ultimo, enquiring if any and what cases of fever have come under my care, which have been occasioned by the want of drainage or other causes capable of producing fever, I beg to state, from an experience of thirty years, during which time I have been the medical attendant of the poor of Hammersmith, that we have always had, at certain seasons of the year, fever prevailing to a great extent among the poor, attributable in a great measure to miasma, produced by a quantity of water which had been left stagnant on the surface of the earth, after brickmaking, and which, in process of time, had become full of vegetable matter. Some years ago this evil had become so alarming that the inhabitants, influenced by the respectable medical men in the neighbourhood, agreed to adopt measures for improving the drainage, and the parish expended considerable sums in so doing, but we have still several places inhabited by paupers without any drainage at all, or what there is so very insufficient that a great quantity of filth of all descriptions is constantly lying on the surface......It appears by the register of sickness and mortality, that we had 104 cases of fever from the 29th of September to the 23d of March, and the greater part of these are certainly to be attributed to causes that might be removed by improved drainage or greater cleanliness." (p. 20.)

Another medical officer delivers himself thus:

"In many parts of this parish a total absence of fever is but of rare occurrence, and it is generally more prevalent in spring and autumn. Although undoubtedly much may be attributed to insufficient drainage, a great deal of disease is produced by the careless and dirty habits of the lower order of people dwelling in many parts of this neighbourhood, who, regardless of all consequences, persist in throwing rubbish and other offensive matters in the streets in front of their houses, which naturally engender much disease." (p. 21.)

Sixthly, the disease is traced to the "lodgment of filth in large cesspools and privies, in situations where the exhalations are destructive of health." The following is the evidence of this:

"You request me," says the medical officer, "in your letter now before me, to describe the nature of such places where fever has most prevailed; to which I reply that fever has been most severe in those courts and alleys where there is no free circulation of air; such as, for instance, Johnson's Change, in Rosemary lane, in which there are about twenty houses, in almost every one of which fever prevailed. The disease first made its appearance there in the month of August last, and on my first visit I found the intolerable nuisance of the overflowing of a cesspool or privy, which continued for some time, there being no sewer to carry off the soil." (p. 21.)

Seventhly. "The situation of slaughter-houses in densely-peopled districts, among narrow streets, and the bad ventilation of these establishments," is commented on in the following letter.

"In answer to your communication of the 27th ultimo, I beg to state that the parish with which I am officially connected comprehends the poorest and most dirty, lowest and worst ventilated, parts of the city of London, chiefly inhabited by the humblest classes of the Irish, and the most abandoned of both sexes; West street, John's court, and Field lane, with the numberless intricate labyrinths and courts, the haunts of prostitutes, pickpockets, and thieves of every description, in which fever seems to have taken up a permanent abode. I have known it to exist there through heat and cold, through wet and through drought, through every variety of weather; and that the district has never been wholly free from it. Owing to the absence of cleanliness, the crowded state of the rooms, six or seven inmates sleeping in one small room, intemperance, the accumulations of dirt and filth that are allowed to take place, all contribute to feed disease and to futilize the efforts of the medical attendant to eradicate it. In addition to this, the number of slaughter-houses that are in the neighbourhood, or on its immediate confines, and the Fleet ditch, the reservoir of all the contiguous sewers, runs underneath these places, above the bed of which many of the houses in the back alleys of Field lane are only a few feet elevated; all these circumstances constitute the constant source of the generation of contagion. The last six or seven weeks we have been called on to attend many cases of typhus fever, of a very malignant character, chiefly attacking the labouring classes, residing in the dirty and most unhealthy portions of our locality. Upwards of twenty cases have occurred within the last three weeks, three of which terminated fatally, two taken into the workhouse, &c. The majority attacked were those who were unable to procure adequate nutriment, from want of employment during the last inclement winter." (p. 22.)

In the eighth place there is an account of the bad effects of certain burial-grounds in crowded districts evinced by the fact that "a court filled with poor people (not forty yards from one of these burial-grounds) was attacked by fever." (p. 23.)

And finally, reference is made to "the want of ventilation in narrow alleys and close courts inhabited by the working classes," in which there is little else than a repetition of what has been stated under the previous heads.

At page 13 of the Report there are some curious particulars of the effects of malaria from some of the causes noticed above—for it appears that the production of contagious fever is only one of the many ailments which may flow from the state of matters to which we have been referring. The following are some "illustrative facts" by Dr. Arnott.

"1. In the field near Euston square, towards Somers-town, now occupied by the commencement of the Birmingham railway, there was until lately, near some very extensive cow-sheds, the meeting of several public drains or sewers in an open ditch, which often overflowed and covered a considerable space with a lake of the most odious filth. In the neighbourhood of this field typhoid fevers were frequent, and in a school of 150 female children in Clarendon square, Somers-town, every year while the nuisance was at its height the malaria caused some remarkable form of disease. In one year it was an extraordinary nervous affection, exhibiting rigid spasms, and then convulsions of the limbs such as occur on taking various poisons into the stomach: more than thirty of the girls were thus affected. In another year it was typhoid fever, affecting an equal number of children; in another, ophthalmia; in another, extraordinary constipation of the bowels, and so forth. Since the covering of the drains all these diseases have disappeared."

Before passing on to the next part of the report, which is from the pen of Dr. Southwood Smith, we would beg to offer a few remarks on the views illustrated by the extracts we have given. We must candidly state our extreme surprise that in these days such meager details as the above should have appeared to the gentlemen who drew up the Report to justify the strong opinion which they express on the origin of contagious fever. If they will but leisurely consider the documents they have selected for publication, it is hardly possible that they can fail to observe —what everybody else must see at once—that the utmost they have succeeded in proving is simply the *coincidence* of fever and the offensive effluvia of filth, while there is not the vestige of anything bearing the semblance of *proof* that the former stands to the latter in the relation of an effect to its cause. Having allowed themselves to prejudge the question, the authors of this Report appear to have entirely forgotten that fever could possibly originate from anything but malaria; and therefore hesitate not to maintain "that by proper sanatory *police regulations*. . .

. . . the typhoid fevers of London and other places might be made to disappear." (p. 14.) No doubt the subject is an involved one: there are many circumstances affecting the class in which fever chiefly prevails, claiming attention in the investigation, among which the offensive state of their dwellings and neighbourhood occupies a very prominent place: yet we think that the philosophical enquirer, with only such materials before him as those on which we are commenting, has no ground for any other conclusion than this, in respect to bad air (malaria) and fever,—that both are common attendants on poverty. How far other, both closer and more constant companions of destitution, may operate in the production of fever we shall have occasion to notice as we proceed; meanwhile we would direct the attention of our readers to those passages in the extracts which prove the existence of extreme privation among the unhappy persons who are described as most liable to fever. Without stopping at present to comment on the many improbabilities which readily present themselves in the conclusions of Drs. Arnott and Kay, we would specially notice, in passing, the difficulty expressed by one of their correspondents, at p. 18, in determining on which of the many peculiar circumstances which affect the poor fever more particularly depends, and to the important testimony of Mr. Evans, p. 25, whose letter is given for another purpose. "In looking over my books," says Mr. Evans, "I find that, in the space of nine months I have attended upwards of 500 pauper cases (of fever), but I cannot trace the disease to any local cause; for we have in the parish of St. George very good drainage through the parish, and very little accumulated filth, with the exception of Falcon court, White street, Noel's court, Hunter street, and Peter street (Mint), but here the disease does not exist more severe than over the parish in general." Of the curious "illustrative facts" of Dr. Arnott, we surely require to say very little: no one knows better than that most ingenious physician and philosopher that an apparent cause may be very far from being a real one, and that the recent removal of a nuisance and the simultaneous cessation of a disease may have no mutual dependence.

The Supplement No. 2 of the Reports is the production of Dr. Southwood Smith. From this we make the following extract:

"The exhalations which accumulate in close, ill-ventilated, and crowded apartments in the confined situations of densely-populated cities, where no attention is paid to the removal of putrefying and excrementitious substances, consist chiefly of animal matter; such exhalations contain a poison which produces continued fever of the typhoid character. There are situations, as has been stated, in which the poison generated is so intense and deadly that a single inspiration of it is capable of producing instantaneous death; there are others in which a few inspirations of it are capable of destroying life in from two to twelve hours; and there are others, again, as in dirty and neglected ships, in damp, crowded and filthy gaols, in the crowded wards of ill-ventilated hospitals filled with persons labouring under malignant surgical diseases and some forms of typhus fever, in the crowded, filthy, close, unventilated, damp, undrained habitations of the poor—in which the poison generated, although not so immediately fatal, is still too potent to be breathed long, even by the most healthy and robust, without producing fever of a highly-dangerous and mortal character." (p. 32.)

One object of Dr. Smith in this and a previous passage is to show that while exhalations from sources in which vegetable matter predominates produce what are known as the marsh fevers, those which proceed from putrefying and excrementitious substances in populous towns produce the continued typhus fevers, because they are the products of animal matter chiefly. A shade of probability or congruity may possibly be supposed to arise from this ingenious account of a presumed adequate difference in the poisons. But even this plausible appearance of completeness in the theory is destitute of any real value. We apprehend that animal matters do not enter so largely into the commissariat of the poor as to leave so formidable an accumulation of relics as shall materially taint the atmosphere in which they live; and we know no process by which the feculent remains of their vegetable diet can be so transformed that the impure heaps which surround their dwellings can be justly esteemed impregnated with a preponderance of animal substances. The passage we have quoted and its context are objectionable also on this ground, that there is a manifest confusion of the known asphyxiating effects of mephitic gases with their presumed capacity to produce typhus fever. If the several statements of Dr. Smith were put into the simple form of the only proposition which they really contain, they would amount merely to this, that exhalations from certain putrescent matters have the power of producing both asphyxia and continued or typhus fever; the former of which is a result familiar to all, and the latter a mere assertion deriving a little hue of probability from its juxtaposition to a known truth. There is a wide difference between the asphyxia which is caused by mephitic gases and typhus fever—a difference which can never be explained, as Dr. Smith attempts to do, by a reference to the diversity in the doses of the poison. We presume that if a few doses of the poison in its less potent shape were sufficient to create typhus fever, *a fortiori* such a quantity of it in a more concentrated form as would be capable of producing a state of asphyxia not ultimately fatal, would, commonly at least, leave the sufferer for days or weeks in the toils of a "highly-dangerous" fever; yet the reverse is the case, as the histories of mephitism amply demonstrate.

A large portion of Dr. Smith's Report is occupied by details of the disgraceful condition of many parts of the Bethnal Green and Whitechapel districts, in respect to filth and defective sewerage; and it would appear that they richly merit his assertion that "it is not possible for any language to convey an adequate conception of the poisonous condition in which large portions of both these districts always remain, winter and summer, in dry and in rainy seasons, from the masses of putrefying matter which are allowed to accumulate." (p. 34.) That this "poisonous condition" is the source of the fever which frequently prevails to a considerable extent in these unions is the doctrine which is maintained throughout, because "from the constant prevalence of fever in these and other districts, it could not be doubted that the poison of fever is constantly generated in these places;" and from the local causes —the depositories of filth, which are described in ample detail. We need not adduce his evidences of the neglected and filthy state of the districts which he has selected for description; they form but a more highly-coloured picture of the same kind as those which we have already noticed. In the following quotation, however, there are some things worthy of a moment's consideration.

"There is evidence, derived from the history of these very localities, that the formation of a common sewer, the filling up of a ditch, the removal of stagnant water, and the drainage of houses, have rendered a district healthy, from which, before such measures were adopted, fever was never absent. This is strikingly exemplified in the present healthfulness of the upper part of the Hackney road, in which an excellent common sewer has been *recently* made, the neighbourhood of which is now well drained. In this part of the district no case of fever is known to have occurred during the present epidemic (1838), although formerly the houses, even in the principal thoroughfare, and more especially the streets, lanes, courts, and alleys adjacent, were the constant seats of fever. A still more striking illustration of this fact is afforded by the altered condition as to the health of the lower part of High street, Aldgate, in the jurisdiction of the city of London. The south side of this street is occupied by butchers, and the slaughter-houses are behind the street. Formerly this place was in an exceedingly filthy condition; at that time fever of a typhoid character was *occasionally* prevalent in all this neighbourhood. About three years ago a common sewer was made by the corporation of London in this street, into which, after incredible trouble, the commissioners succeeded in inducing the butchers to open drains from the slaughter-houses and the dwellings around. Even now the blood and filth from the slaughter-houses lie sufficiently long on the surface to produce an offensive odour; but on account of the excellence of the drainage, the same particles of matter do not lie sufficiently long to putrefy. Fever has been comparatively absent from this neighbourhood ever since the opening of these drains. Dwellings thickly crowded with inhabitants, stand all around the slaughter-houses, yet here, where the materials for the production of the worst forms of fever are most abundant, scarcely a case has occurred even during the present epidemic. On the other hand, in the passages, courts, and alleys, on the very opposite side of the street, from the houses of which there are no drains into the common sewer, fever of a fatal character has been exceedingly prevalent. I have myself *very recently* attended several families in these courts labouring under the worst forms of spotted fever; but I have neither seen nor heard of a case on the opposite (the south) side of the street; whereas there is hardly any part of Bethnal-green or Whitechapel in which fever has been more prevalent or fatal than in the streets, courts, and alleys which go off from High street, Whitechapel, continuous with High street, Aldgate, to which the before-mentioned sewer does not extend. In the streets, courts, and alleys just adverted to, which branch off from the main street of Whitechapel, there is either no drainage at all, or what there is is superficial and exceedingly imperfect." (p. 35.)

Without adverting to the proofs which exist of the incapacity of such effluvia, as are here specially referred to, to originate contagious fever, we take the passage simply on its own terms as professing to detail the *experimentum crucis* in respect to the agency of malaria. We object, first, to the insufficiency of the facts. Every one who has attended much to the local histories of fever is familiar with the circumstance that in the same town the disease is very liable to shift its seat, not only during the persistence of one epidemic but in successive epidemics, so that the region specially affected by one of them may enjoy even a total exemption from its successor, though no changes have occurred in the locality during the interval. Dr. Smith gives us no precise statement of the history of the south side of High street, Aldgate, in respect to such exemptions, but he admits that while in its filthy state fever was only "occasionally prevalent" there. Why not *always*, since "formerly this place was in an extremely filthy condition?" There must surely be a something else than malaria necessary to generate fever, if, the local impurities remaining the same, fever was only occasionally present. The recent nature of the experiment in both the Hackney road and the High street in question deprives the statements of any degree of weight in respect to the point at issue.

In the second place, the accounts given by Dr. Smith, if they prove anything in regard to the effects of the improved sewerage, prove rather too much. They prove that a common sewer may deprive fever of its contagious power. We have the remarkable phenomenon of "dwellings thickly crowded with inhabitants," and these inhabitants the poorer classes, rendered incapable of harbouring the contagion of a highly contagious disease in consequence of the "excellence of the drainage!" The new sewer, if the recent exemption is to be traced to it, proves just as much in respect to this as to the former conclusion. How much more just it would have been to have ascribed the late freedom of the locality from fever, as well as the former occasional exemptions, to the non-introduction of contagion, we leave our readers to judge; for even Dr. Smith does not exactly hold that good drainage will prevent the operation of contagion, as appears from his expressions at p. 36, "fever will sometimes spread among the inmates even in the best drained, the best ventilated, and in all respects the best regulated houses," (workhouses.)

After concluding his account of the filthy state of Bethnal-green and Whitechapel unions, and of the prevalence of fever in those districts, Dr. Smith observes, "The previous statements connect in the clearest manner the prevalence of fever with poisonous exhalations arising from putrid vegetable and animal matter" (p. 43); and in further illustration of the same opinion he quotes at length from Pringle's Observations on the Diseases of the Army, an account of the malaria and its effects which were witnessed in Flanders. The author appears, strangely enough, to confound the origin of the remittent fevers of the Low Countries, which are incommunicable diseases, with that of typhus, and to draw an argument in favour of the malarial origin of the latter from what has been amply acknowledged in respect to the former; a course about as justifiable in medical logic as it would be to trace measles to almonds or oysters because these can produce the nettle-rash, which ranks also among the exanthemata. It is astonishing to us who know Dr. Southwood Smith's great powers of mind and his acute and logical spirit, that he could have allowed such a passage as the following to drop from his pen. The only way we can account for this and several other things in the work before us is by supposing that the enthusiasm of philanthropy and a vivid imagination have secretly jumped to desired conclusions, while the cool and calculating eye of philosophy was closed in slumber.

"The room of a fever patient, in a small and heated apartment in London, with no perflation of fresh air, is perfectly analogous to a standing pool in Ethiopia, full of the bodies of dead locusts. The poison generated in both cases is the same; the difference is merely in the degree of its potency. Nature, with her burning sun, her still and pent-up wind, her stagnant and teeming marsh, manufactures plague on a large and fearful scale. Poverty in her hut, covered with her rags, surrounded with her filth, striving with all her might to keep out the pure air and to increase the heat, imitates nature but too successfully; the process and the product are the same; the only difference is in the magnitude of the result." (p. 33.)

How all this has been determined we need not ask, because we are sure that Dr. Smith would be puzzled to reply; but we must record our regret that in these days of laborious research, loose notions, like the above, should emanate from men of high reputation and deserved influence, as they cannot fail to have the effect of hindering the progress of truth. Dr. Smith illustrates and supports his doctrine of the malarial origin of continued fever, by referring to facts which relate merely to periodic fevers; and he maintains the identity of the "fever-poison" of this country with the poison of the plague; wherefore, on the principle that things that are equal to the same thing are equal to one another, plague and ague are generated by the same poison!

Quitting these speculations, which carry us a century back in etiology, we would observe, that the direct evidences which may be adduced to prove the insufficiency of the exhalations from putrefying organic matter to generate contagious fever are neither few nor inconclusive; and that even if such evidences were defective there would, notwithstanding, remain the necessity of proving that such malaria is the source of fever, in preference to other circumstances existing in the same localities, and its poisonous effects in respect to fever not a mere fancy which the long habit of repetition has dignified with the importance of a dogma. The many facts which have been accumulated in evidence of the innocency of putrescent exhalations must, partly at least, be known to those who contend for the malarial origin of fever; we should therefore have expected that instead of contenting themselves with adducing instances in which malaria and fever coexisted, they would have taken some pains to expose what inaccuracies they perceive in the opposite details. But this they have omitted to do.

There are some old and some new histories at utter variance with the conclusions and hypothesis of the Reports on which we have been commenting. Although it has been justly remarked, that a fact never becomes old, that is to say, superannuated, we shall pass by the testimony in exculpation of malaria as the source of fever which is yielded by the exhumations at Dunkirk in 1783, and at Paris in 1786, as well as the many details collected by Chisholm and Bancroft, all of which are well worthy of being considered; and shall introduce to our readers, what may be new to many of them, the researches of Parent-Duchâtelet, the notice of whose admirable work, in our Fifth Vol. (p. 447), was, we regret to say, much too imperfect. Fifteen years of a life which terminated prematurely for the science to which he had devoted his uncommon energies, witnessed his unwearied application to his favorite study, while they furnished him with ample materials for the training of a mind deeply imbued with the true spirit of induction. We find in the essays of Duchâtelet, almost everything we miss in the pages of the "Report." We find enquiries conducted on a comprehensive scale, circumstances noted, weighed, and compared, with a care and precision commonly confined to the experimental sciences; and, therefore, conclusions that claim our confidence and assent.

The Essays of Duchâtelet embrace many subjects connected with the public health, and among them we find some to our present purpose. The immense reservoir of impurities at Montfaucon can scarcely be surpassed in fitness for an enquiry respecting the effects of putrid effluvia.

"The influences of this *voirie*," says Duchâtelet, "ought to increase equally with the quantity of matter which is deposited there; a multitude of facts prove, and reasoning alone may suffice to demonstrate, that the intensity of offensive emanations which proceed from any place is always in proportion to the quantity of the matters which furnish these emanations: at present, those which proceed from the '*voirie*' of Montfaucon, are constantly insupportable in a circumference of two thousand yards; the winds carry them sometimes, in all their intensity, to more than four thousand yards; and it appears from accounts collected by the commission charged with ascertaining the ravages of cholera in the rural districts, that certain atmospherical conditions, rare it is true, conveyed them to the extent of eight miles. Could it be otherwise, since the basins alone of this reservoir contain 32,800 yards of surface, without reckoning twelve acres occupied by dry matters and the curriers' yards; since from 230 to 244 cubic yards of the products of the 'fosses d'aisances' are carried thither daily; and since the greater part of the bodies of twelve thousand horses, and from twenty-five to thirty thousand small animals are left to rot on its surface!" (tom. ii. p. 287.)

More in detail we have an account of the curriers' yards situated on the borders of the general receptacle.

"The nature of the operations which are practised in those yards, the fetid emanations which proceed from them, and the disgusting and destructive animals of which they favour the increase, have always made them to be considered as dangerous places which ought to be far removed from habitations. .... In reality nothing can be compared to the fetid effluvia (l'infection) produced by these establishments; everything there announces negligence and barbarism; their aspect alone induces one to draw back with horror; and one asks himself, on beholding them, if he be actually in the nineteenth century, and at the entrance of a city which makes pretensions to be the capital of the civilized world." (tom. ii. p. 123-4.)

Into this central establishment for the slaughter of horses, it is calculated that 12,775 of these animals are annually brought. In the "chantiers d'équarrissage," the bodies of these animals undergo a multiplicity of operations; the hair, the hides, the flesh, the bones, the entrails, all are more or less the subjects of labour and the sources of effluvia.

"Let one figure to himself what may be produced by the decomposition of heaps of flesh and entrails abandoned, for weeks and months, in the open air and to the heat of the sun, to spontaneous putrefaction; let him add, in thought, the nature of the gases which can arise from the heaps of carcasses which remain covered with much of the soft parts; let him further add the emanations furnished by a soil, which for years has been drenched with the blood and fluids of animals; those which emanate from this blood itself which, in both of the yards, lies on the pavement without being able to escape; those, in fine, of the kennels of the gutspinners and dryers in the neighbourhood; let one multiply as much as he pleases the degrees of stench, by comparing it to that which every one of us has been enabled to perceive on passing near the bodies of animals in decomposition, which it was necessary to encounter, and but a feeble idea shall be formed of the truly repulsive odour which emanates from this sink, the most offensive that it is possible to imagine!" (tom. ii. p. 222.)

Surely these descriptions prove the existence of a malaria, compared to which the effluvia of a slaughter-house or a common sewer are as nothing; surely in such a place, if anywhere, fever must haunt the habitations of men, and fetter its victims in the helplessness of the most malignant forms of typhus. Let us see what Duchâtelet has ascertained on this point.

"If we ask the master *équarrisseurs* and the workmen (knackers), one after the other, they reply to us, that they are never ill, and that the emanations which they continually breathe, far from being hurtful, contribute to their good health: this evidence is assuredly important, but it does not suffice. Let us seek for proofs more convincing. If we examine them, we shall see that they bear all the characteristics of a health the most flourishing, and that in this respect they resemble much our butchers. We are not the only persons who have made this observation, as proved by the passage we have quoted from a Report made in 1810 by MM. Deyeux, Parmentier, and Pariset, on the yard which existed then at the Gare. These gentlemen speak of the surprise they felt at the brilliant health of the woman named Fiard, and her five children, who laboured the whole year in the yard, and slept in the same place, where it was impossible for the members of the commission to penetrate, on account of the extreme fetor which it exhaled. All, however, do not attain the robustness which is common to the majority; some remain thin, though preserving good health. We have made this remark both on males and females. . . . . Are the chances of longevity less favorable among these than among other artisans? Everything appears to prove the contrary. We see many *équarrisseurs* who are sixty and seventy years old, and who are perhaps the strongest and the most active of those who work in the yards of Montfaucon. We have taken precise notes respecting their fathers and mothers, and have learnt that they all died at a very advanced age, and almost always exempt from the infirmities of old age. . . . . These singular facts, so much in opposition to what has hitherto been published respecting the influence of putrid exhalations, are confirmed by the long experience of MM. Damoiseau and Huzard, and especially by the latter, who for sixty years has not ceased to have almost daily intercourse with the *équarrisseurs.* It may be said that these workmen, born so to speak in the trade, and all the issue of parents who have exercised it, have lost the capacity of being influenced by putrid emanations, which yet exert all their activity on others: we reply to this objection by the following facts: The strangers who come often, or even every day to the yards, and who remain there a longer or shorter time, are not incommoded." (tom. ii. pp. 228-9.)

This statement is illustrated by the fact that new workmen occasionally employed are not remarked to be more subject to disease than the others. Duchâtelet made enquiries also among the quarriers and plaster-makers of the vicinity, who are exposed to all the influences of the yards; and though all agreed respecting the discomfort produced by the effluvia, yet none of them accused these as affecting the health of their numerous workmen, although they labour while the putrescency is at its greatest degree of intensity, during the heats of summer. *Apropos* to these details M. Duchâtelet introduces a notice of certain other sources of putrid animal exhalations which exist in Paris. Every year nearly two hundred exhumations are performed at one of the cemeteries, in order to transport into new grounds, or tombs, bodies temporarily deposited in particular vaults; and although these exhumations are carried on at all seasons of the year when the bodies have been several months dead, and putrefaction going on with activity, yet it has "never hitherto been remarked that the least accident has happened to the workmen engaged in these labours, which are the more toilsome, and ought to be the more dangerous that they are obliged to breathe, in the vault itself, the emanations which have been shut up for a long time in a narrow space." (tom. ii. p. 230.) He brings also to the support of these facts the observations of MM. Guersent and Labarraque, who have proved that the manufacturers of gut-strings enjoy the best of health, though living in an atmosphere of effluvia, and continually in contact with intestines undergoing a lengthened maceration.

It would appear that there have been in Paris, as well as in London, functionaries abundantly disposed to confide more in their organ of smell, than in their reasoning faculties, while investigating the condition of the public health, and the sources of disease, of which the following deliberate determination, expressed in a Report for 1832, and quoted by Duchâtelet, affords an instructive example: "As for us, in spite of all the reasonings of tradesmen, and all the logic of science, our spirit refuses to believe that establishments so loathsome as those of Montfaucon, do not present any cause of insalubrity." (p. 237.) So much for the indomitable spirit of prejudice.

In the same volume there is an account of the effects of the atmosphere of the Parisian dissecting-rooms, on the health of those exposed to it, of which we gave a pretty detailed notice in the article already referred to. (Br. and For. M. Rev. V. p. 452.) We shall here merely repeat the result of M. Duchâtelet's own experience of five years' connexion with the dissecting-rooms: he says, "We can affirm that the number of those who have become ill in a serious manner, during the course of their anatomical studies, has not been one in a hundred; now, when of 100 individuals, subjected for six months to the action of any influence, ninety-nine experience nothing; it appears to us, that in good logic, the exception furnished by the hundredth ought to be considered as null." (tom. ii. p. 36.) These will be esteemed no despicable facts by those who have spent some hours in a Parisian dissecting-room; and by those who have not, some faint conception of their atmosphere may be formed, when it is stated that, in the rooms of Clamart, from 1200 to 1400 bodies are annually dissected, and that no particular attention is bestowed on cleanliness. There are many other accounts of the exposure of persons to the effluvia of decomposing animal matter, contained in Duchâtelet's work, of which we can make no special mention. Before quitting the consideration of these animal effluvia, however, we would briefly notice a passage illustrative and explanatory of the opinions entertained on the cause of fevers connected with military operations. While commenting on certain objections urged against the salubrity of a particular manufactory, Duchâtelet observes, "You speak of the typhus which the projected manufacture might produce; you say that in 1814 fevers and typhus were seen to follow the disastrous battle of Paris. We have seen the typhus, in 1814, accompany the French army in its retreat, and make dreadful ravages in it; we have seen it arrested spontaneously in April of the same year, and since that time, it has not come to our knowledge that any one has observed it in an epidemic manner near Montfaucon, or anywhere else; as to the battle of Paris, if the emanations from the bodies could give origin to typhus, it is in the villages of Belleville, Pantin, and Prés-Saint-Gervais, that it should have been noticed, because the most deadly actions took place within their walls, or in their vicinity." (tom. i. p. 47.) The action occurred on the 30th of March, 1814, and of the results which might be presumed to affect the health of the neighbourhood, it is stated that "4000 horses were collected in the neighbouring plains; they were all accumulated at the same place;.... for ten days they exhaled a noisome stench; however, they were not injurious either to those who directed the operation (of burning them), or to the numerous workmen who seconded them." (tom. i. p. 48.) At the villages specified above, it has been ascertained from the registers, that fewer deaths occurred in April, 1814, than in the same month of the year before and the year after. These facts afford good ground for suspecting that other causes should be blamed for the prevalence of fever in bodies of troops than the infected air of battle-fields, to which it has, from time to time, been referred; and, indeed, the other accounts which we have quoted from Duchâtelet strongly attest the insufficiency of such a state of the atmosphere to produce the effect ascribed to it.

In the manufacture of a particular manure from the contents of the "cabinets d'aisance" and sewers, deposited in the general reservoir at Montfaucon, exposure to fetid exhalations is so great, that those occupied in this employment should of necessity be the subjects of any evil effects which such exhalations are capable of producing. This manure is termed Poudrette, and is neither more nor less than dried ordure. Circumstances led Duchâtelet to make enquiries respecting the health of the persons engaged in this disgusting business; and the following is a summary of his observations.

"Before engaging in the researches, of which I am about to state the result, I was so persuaded that the 'voirie' of Montfaucon, would furnish me with the *data* which were necessary, that I regarded the question as already settled; I confess that my surprise was great, when instead of encountering a population frail and languid, as I expected, I did not see a single workman who did not present all the exterior signs of the best health. I was at first tempted to attribute to habit this faculty, which they had to remain unscathed in the midst of fetid odours and emanations, which I regarded as very dangerous; but it was easy for me to see that habit went for nothing, since the new workmen, whom they received every day, are not more affected after three or four weeks than those who have laboured there ten and twenty years; moreover, there exists, among the workmen of the 'voirie' an opinion that the emanations which proceed from it, far from being noxious, have, on the contrary, a salutary influence on the health, that they protect from epidemics and cure many diseases." (tom. ii. p. 273.)

Though inclined to question these latter conclusions, while resting on the authority of these workmen alone, because they might possibly be interested in concealing the truth; yet, being open to conviction from adequate testimony, he could not but yield credit to the facts when attested also by the agricultural population, the carriers, and manufacturers of plaster, resident in the vicinity, although directly opposed to his previous opinions. It is necessary to add to the foregoing statements an account of the manufacture in question, and of the locality in which it is conducted; the details of which are of a nature which throw far into the shade the descriptions of impurity and loathsomeness which we had previously perused. The "voirie" of Montfaucon, is composed of six basins on successive elevations. In the two highest are deposited the contents of the privies of the city on their arrival at the "voirie;" the four others are but receptacles of the liquid matters which flow from the upper basins. The average quantity of matter transported annually to these basins, amounts to above 16,000 loads. The matter in the highest basin, which, from the partial separation of the more liquid substance, acquires a pasty consistency, is that which is dried for the purpose of making one sort of "poudrette;" for this purpose it is withdrawn from the basin, is spread on certain neighbouring pieces of ground appropriated to the purpose of drying it. When sufficiently dry it is raised in heaps eight or ten yards high, and from sixty to eighty long. In this form it remains for a long time, sometimes for several years; and, finally, it is reduced to powder. In this last state it presents the appearance of a dark gray earth, of little weight, unctuous to the touch, friable, and exhaling a nauseous odour. Another kind of "poudrette" is made from the liquid matter of the basins, which consists simply of urinary substances, with more or less of the matter already described. That these several processes are eminently calculated to afford the amplest scope for the exhalation of every noxious principle which filth can contain, will scarcely be questioned; yet it is in an atmosphere offensive beyond comparison, in consequence of these exhalations, that health and remarkable exemption from epidemic diseases have been ascertained to exist.

Our readers need not apprehend that we are about to advocate the salubrious qualities of fetid effluvia, or to defend the neglected and filthy condition of many parts of our large towns. We shall by and bye show that the freedom from epidemic diseases which the workmen at Montfaucon enjoy, can be traced to another peculiarity of their situation, and that the details which we have quoted, afford in reality no plea for nastiness; but *il ne faut pas faire le diable plus noir qu'il n'est.*

We cannot quit the work of Duchâtelet without noticing his account of the stream of Bièvre, which traverses a part of Paris, and its effects upon the public health. He informs us that it is difficult to form any conception of the state of this stream in summer, when its bed is nearly dry; its breadth is from eight to ten feet, and it varies in depth from seven feet to one or two; and scarcely has its mud been rendered somewhat dry than it cracks and sends forth exhalations "insupportable to those who pass in the vicinity, and much more so to those who dwell on its margin." This state of matters begins to be noticed above Gentilly; but is much more remarkable in Paris, "where the mud, entirely composed of the *debris* of animal substances, swells when it is dry, puffs up, and bursts, precisely like dough subjected to fermentation." (tom. i., p. 128.) In consequence of the great exhalation of gases from the putrefying mass, meat cannot be preserved during summer more than eight or ten hours; plate and the "batterie de cuisine" are altered and tarnished, for it is chiefly where the stream flows among the houses, and ventilation is prevented, that the evil is the most distressing. "One would conceive, at the first sight, that such a focus of putrescency, traversing a quarter inhabited by 30,000 individuals, for the most part in poverty, and crowded together, should produce serious diseases, or at least give to this population an appearance of languor and debility as the predominant character of their constitution; such is at least the idea generally entertained in Paris respecting this part of the suburb of Saint Marceau. . . . . . . . It is not without lively satisfaction that we are enabled to reassure the inhabitants as to the results of the influence which the exhalations of Bièvre have on the health of those who are exposed to them, whether temporarily or continually." (pp. 129-30.) Although prejudiced against the stream and its influences when he began the enquiry, he found it impossible to establish any difference in the physical constitution of those who resided on its borders and of those who lived in other quarters. The register of a dispensary existing in this district for twenty years exhibits no peculiarity in the maladies prevailing among the inhabitants, nor anything in the diseases in general different from those of other quarters. The truth of these conclusions is attested in the strongest manner, and established by enquiries and observations made in every season of the year. To the same purpose is the account given of the health of those engaged in cleaning the common sewers of Paris, often a fearful business it would appear; but we have not space for any details on this head.

The publications which relate to the "fetid irrigations" practised in the vicinity of Edinburgh, do not for the most part contain anything very clear in respect to their properties, with the exception of evidence that they are abundantly offensive. It seems that on two sides of the city a considerable extent of ground is rendered highly productive of a luxuriant grass by means of certain common sewers.

"Without affecting precision, we may say that the meadows to the west extend two miles in length and one in breadth, and contain about two square miles, while those to the east of the city may be about two miles long by half a mile broad, and contain perhaps one square mile. The sum total is three square miles, that is, 1920, or about 2000 acres, equivalent to 87,120,000 square feet of poisonous swamp. This, then, is the surface covered with the drainage of the city, where it is spread out in small currents, which sink into the ground till it be completely saturated like a sponge. The sun, acting on the ground so prepared, decomposes the animal and vegetable matter there deposited, and gives rise to all those effects formerly enumerated; while, whatever wind may blow, the exhalations are wafted from one or the other system of drainage to every part of the town." (Papers relating, &c., p. 22.)

This very reprehensible system of irrigation has been gradually increasing in extent for the last thirty years, and now amounts to so great a degree as to become extremely offensive in its effects on a considerable portion of the town, especially on its eastern skirts. Contagious fever has been gradually on the increase also in Edinburgh for above twenty years, and nothing is more natural than that the two evils should be regarded somewhat in the light of cause and effect. We need not enter into the evidence which is adduced to prove the insalubrity of the irrigations, the more especially as nothing appears to be proved but that disease exists within the range of their exhalations, and that if offensive effluvia from decaying animal and vegetable matter be capable of producing putrid diseases, the vicinity of those fetid marshes ought to present ample testimony to the fact. On this subject little is adduced beyond mere opinion, and where facts are stated they are much of the inconclusive character of those contained in the reports already referred to.

Very different are the facts adduced on the opposite side (for the subject has been stoutly contested), particularly in respect to the connexion of the irrigations with fever. The principal facts are the following: Fever attains its greatest amount in winter when the irrigations are discontinued, and in summer, when the irrigated grounds are sending forth their effluvia in such abundance and intensity as to produce sickness and other disagreeable effects on those passing in their vicinity, fever is very generally at an extremely low ebb. The greatest number of cases of fever does not occur in these districts more immediately in contact with the marshes, a circumstance which is attested by the records of the Fever Board for four years prior to 1838, the particulars of which are contained in page 38 of "An Examination of the Statements, &c.," and has been confirmed to ourselves by information of a corresponding description derived from the Records of the Royal Infirmary of Edinburgh. "Fever is not confined to any particular locality in Edinburgh, with this exception, that it is generally most prevalent where the inhabitants are poorest." (An Examination, &c., p. 25.)

It may be said that these statements in reality prove nothing in respect to the question at issue, because typhus fever is not maintained to be the offspring of marshes. These irrigated lands are, however, not mere marshes; they are rendered fetid, not by the decay of the vegetable products of the soil, but by the contents of the city drains and sewers—the very matters to which so much is ascribed in the generation of fever.

We sometimes hear and read of contagious fever being referred to the exhalations from putrefying mud on the margins of rivers, ditches, and ponds, and the fever of the metropolis we observe to be referred to this source from time to time. We need scarcely remark that this is the very source to which the worst forms of the periodic fevers are commonly referrible, as is familiar to those who are acquainted with the history of those diseases; and if the same substance can also produce continued fever we should expect that wheresoever the latter prevailed in connexion with an apparent cause of this kind, we should have a certain proportion of the periodic fevers likewise, which are unquestionably of malarial origin, and it is probable that the continued and periodic fevers, if traceable to the same source, would correspond in some degree in their frequency. In the Reports on the sanatory state of the poor in the metropolis, there is notice made of the intermittent as well as of the continued fever to which certain districts are liable; and it appears that there is no relation between the two of that kind which we should expect to exist if they owned a common origin. Thus, intermittent fever is most abundant in Poplar, where the Report shows it to have existed in the proportion of one in every seventy-five of the paupers, while continued fever bore the proportion of one in fifteen; and in Kensington, the intermittents amounted to one in seventy-one of the paupers, continued fever to one in twelve. In St. George in the East continued fever was as high as one in eleven of the paupers while there were no intermittents; in St. George the Martyr's there were only three cases of intermittent fever to 1247 of continued; and in Lambeth, the proportion of intermittent fevers was one in 526, that of continued fever having been one in five of the pauper population. The conclusion to which these facts lead is obvious.

The same conclusion may be attained by referring to histories of the epidemic prevalence of periodic fevers. Dr. Hamilton, in his account of the fevers epidemic at Lynn Regis towards the close of the last century, alludes to several cases of continued fever as having occurred and put on the form of "putrid, malignant, fever," but, he observes, "these were all attacked in the beginning with the reigning epidemic, the remitting fever." In the account which Caillard has given of the epidemic which occurred in the vicinity of the Canal de l'Ourcq, near Paris, in 1810, and subsequent years, there are some circumstances narrated which possess no inconsiderable interest in reference to enquiries respecting the operation of malaria variously compounded. "In the route from Paris to Pantin," says he, "exposed on the one side to the miasmatic emanations of the canal, and on the other to the putrid effluvia of the '*voiries*,' the diseases were numerous, almost all serious and obstinate. ........ This disastrous effect of the union of putrid effluvia with marsh miasmata was especially evident in one part of this route termed the Petit-Pont hamlet, inhabited by a currier and a gut-spinner, the putrid waters from whose operations are prevented from escaping by the banks of the canal, and exposed before the draining to the emanations of a large marsh. This hamlet was so unhealthy, that of twenty-five or thirty inhabitants, I visited above twenty seriously affected, of whom five died." Other similar examples are described of localities exposed to the combined agency of animal and vegetable miasma, producing the more serious class of the prevalent disorders, as at Bobigny, Noisy, and Pantin, the two former containing ponds in the midst of the houses, which were "the receptacles of the impurities of these houses, and of the cattle connected with them," and the latter both in the neighourhood of marshes, and exposed to the effluvia of Montfaucon, and of the putrid manure used on the grounds in the vicinity. Yet the fevers which prevailed in those places were always of the intermittent and remittent types. "When the disease was treated by reiterated purgatives and emetics, it became very easily continued, and had ordinarily a fatal termination on the fifteenth or twenty-first day." Before it can be supposed that these last were cases of the continued fever, to which we are so much exposed in this country, it will be necessary to show that the contagious continued fever assumes at any period the character of a remittent, a disease, by the bye, which has entirely disappeared from London, where the continued form has lately been so prevalent. To the mere circumstance of continuance in fever too much importance is ascribed; a single bloodletting in the cold stage not unfrequently converts a tertian into the continued type; yet the fever does not on that account cease to be a marsh fever, nor does it become a contagious typhus fever.

Among those who contend for the malarial origin of fever two opinions may exist in respect to the nature of the malarial "fever-poison:" to wit, that it is different from the matter of contagion, or, that it is identical with it. There are very serious difficulties in the way of both opinions. If the former be adopted, it is necessarily maintained that the malaria can produce a disease, in the course of which a substance is elaborated quite unlike the original, yet capable of producing the same complicated series of effects; a doctrine to which it would be difficult to produce a parallel in the science of medicine. On the other hand, if it be held that the malaria and the principle of contagion are one and the same in nature and properties, how happens it that in the several instances we have adduced, communities of men have lived, and continue to live, in an atmosphere loaded to the utmost pitch of endurance with the exhalation of putrescent organic substances, without any peculiar liability to fever, while, as is well known, no class of men can for any length of time be exposed to a similarly concentrated contagion, without exhibiting ample evidence of the evil that is among them, in the abundance of its victims? Either there is no "fever-poison" in the malaria of putrefying organic remains, or it is unlike the substance of contagion. The one will produce disease in twenty-two out of every twenty-three exposed to it in no uncommon way, according to the observations of Haygarth, while the other, existing in unusual abundance and intensity, displays no trophies of its asserted power.

We would call particular notice to the instances adduced by Bancroft, Chisholm, and Duchâtelet, of the absence of contagious fever, in circumstances eminently calculated to produce it if putrid effluvia possess such a power, because we perceive, not only from the Report on the sanatory state of the poor in London, but from the recent Parliamentary Report on the Health of Towns, which we have had an opportunity of but hastily glancing at, that there is a tendency among those who have given evidence on the state of the public health, especially in respect to fever, to regard too exclusively the local nuisances in the neighbourhood of the poor, to the neglect of their condition in respect to food, clothing, and employment. Doubtless, allusions are occasionally made to these circumstances, but in a way, generally, which conveys the impression that they have attracted but little notice, and that a merely secondary importance is, at the most, to be ascribed to them in contributing to the prevalence of fever. The proofs which exist of the incapacity of malaria under circumstances of peculiar aggravation to generate fever, as detailed by the authors to whom we have referred, are to be refuted neither by a heedless neglect of them nor by volumes of mere reiteration of a contrary opinion.

We are far from being certain that the best plan has been adopted for gathering comprehensive and accurate statements in regard to the origin and prevalence of fever, by applying to a multitude of individuals for the results of their observations, and the opinions they have founded on them. Few are well fitted for enquiries of this nature, and fewer still, among practitioners of medicine at least, have that undisturbed leisure from professional occupations, so essential for following out a system of deliberate and persevering research, which alone is adapted to yield the materials proper for the elucidating subjects so complex in their relations, and demanding so much careful analysis. The particular merit of Dr. Alison's pamphlet, on the Management of the Poor in Scotland, where reference is made to the causes of fever, consists in the just and philosophic consideration which is extended to the circumstances in which the question is involved, not merely as they have presented themselves in the course of his individual experience, but as they are known to exist in the history of the country and of science. An experiment, so to speak, thus conducted on a grand scale, is of immeasurably greater value, and more worthy of confidence than a thousand little operations, which, owing to their very smallness, are liable to uncertainty and error from many incidental causes. The particular nature and object of Dr. Alison's work do not admit of his entering into lengthened details of the origin and propagation of fever; but we feel assured that he has taken the right method of determining those momentous questions, by instituting a comparison of the different circumstances of the poor in those parts of the country which are the least affected with fever, and in those where fever is the greatest scourge.

Destitution, the want of proper nourishment and clothing more especially, has been often asserted to be the grand basis of epidemic typhus fever; and, therefore, this fundamental doctrine of Dr. Alison's views, in respect to the prevalence of that malady, is neither new nor uncommon. But there is much of the merit of originality, and of the raciness of novelty, in the manner with which the doctrine is handled by Dr. Alison, and in the range of his illustrative argument. In so far as fever is the subject of the work, the proposition which Dr. Alison endeavours to demonstrate is the following: "The existence of epidemic fever in any great community, particularly if there be neither war nor famine to explain it, becomes a most important test to the legislator of the destitute condition of the poor, and, as I shall endeavour to show, of the deficiency of the funds which, in a better regulated state, are applied to their support." (p. 18.) From this passage it may be gathered that Dr. Alison is no advocate of the influence of malaria in generating fever. His views on this subject are more fully declared in the following passage:

"In the appendix to the Fourth Report of the Poor-law Commissioners, it is stated by Drs. Arnot, Kay, and Southwood Smith, that the malaria arising from putrefying animal and vegetable matters produces typhoid fevers. Although I highly respect all these gentlemen, and approve of the practical inference which they draw from that opinion, so far as it goes, because I have no doubt that vitiated air, like all other causes which weaken the constitution, favours the diffusion of fever, yet I cannot subscribe to their opinion, that this cause is of itself adequate to the production of contagious fever. And if, trusting to that opinion, the public authorities should think it sufficient, in any situation where contagious fever is prevalent, to remove all *dead* animal and vegetable matter, without attempting to improve the condition of the *living* inhabitants, I am confident that their labour will be in vain. The true specific cause of the contagious fever, at least in Edinburgh, certainly does not spring from anything external to the living human body." (p. 11.)

Dr. Alison refers to a paper in the Edin. Med. and Surg. Journal for 1828, for the more extended statement of his opinions, and the evidences in support of them. In that article, and in his present work, the author confines himself to the denial of the malarial origin of fever and to the illustration of its propagation by contagion. He recognizes elsewhere in the facts and observations collected by Chisholm and Bancroft, evidences that the effluvia of putrefying organic substances are not the sources of contagious fever; and in the mode in which the disease spreads through a community he perceives chiefly the operation of contagion. Prominently as destitution stands in the work of Dr. Alison among the causes of fever, he gives it, almost exclusively, the secondary rank of a merely predisposing cause, as in the following passage:

"It is not asserted that destitution is a cause adequate to the *production* of fever (although in some circumstances I believe it may become such); nor that it is the sole cause of its extension. What we are sure of is, that it is a cause of the rapid diffusion of contagious fever, and one of such peculiar power and efficacy, that its existence may always be presumed, when we see fever prevailing in a large community to an unusual extent." (p. 10.)

The following extracts from Dr. Grattan's account of fever in Ireland, in 1818, are adduced in illustration of the circumstances in which destitution may generate fever:

"'Next to contagion, I consider a *distressed state* of the general population of any particular district the most common and most extensive source of typhoid fever; whether this has been the result of war, or been produced by the more gradual progress of domestic misfortune.........The present epidemic is principally to be referred to the miserable condition of the poorer classes in this kingdom; and so long as their state shall continue unimproved, so long will fever prevail, probably not to its present extent, but certainly to an extent sufficient to render it at all times a national affliction.........In crowded cities, especially when much poverty prevails, the inhabitants, listless and desponding, become inattentive to cleanliness in their persons and habitations, their contracted means compel numbers to reside in the same dwelling, and their apartments are in general filthy and ill ventilated. In winter, in consequence of the want of fuel and of sufficient clothing, any aperture is closed through which the air might procure admission. In these circumstances the contagion of fever is often developed, and then almost every individual within the sphere of its operation, and predisposed by the debilitating effects of mental anxiety, is attacked by the disease.'" (p. 11.)

This is very nearly the same opinion of the origin of fever as that which has been expressed by Dr. Ferriar, in his Medical Histories, who specifies the following as the principal circumstances which produce the peculiar poison of fever: "1st. Want of fresh air. 2d. Deficient or improper diet. 3d. Want of cleanliness, and chiefly want of proper removal and change of clothes. 4th. Anxiety and depression of spirits." (vol. i. p. 242.) Under these circumstances it is presumed that the poison of fever is generated within the living body.

Doubtless there must be great difficulty in affording anything like demonstrative proof of the actual origin of fever from these causes alone. Contagion is a principle which is naturally suspected to lurk in the localities which have been once affected with fever; and as yet so little is known of its qualities and of its power of maintaining its existence, that every difficulty in the origin of fever is by some as readily resolved by a reference to contagion as by others to malaria. That this opinion is correct in the vast majority of instances it were folly to deny; but that it is correct in every instance it were, we apprehend, nearly as unwise to maintain. Smallpox is commonly made the subject of comparison in questions of contagion. It is concluded to be incapable of being generated anew; and contagious diseases which correspond in the essential features of their history and prevalence with smallpox, may, perhaps justly, be considered as alike in that respect. There is no such correspondence, however, between fever and smallpox. Wherever disaster and privation exist for any considerable time, affecting large communities or bodies of men, fever is a too certain attendant; and hence, times of scarcity and of commercial distress, and reverses of fortune in military affairs, have been esteemed, from ample experience, the periods peculiarly favorable to epidemics of this kind.

We think that there is much evidence in Dr. Alison's work in support of the conclusion to which he has arrived, that the prevalence of contagious fever is "an indication and test of much previous privation and suffering" among the poor.

"Having been," says Dr. Alison, "for many years past a witness of great and, I am sorry to add, of increasing sufferings among the poor of this city (Edinburgh), having satisfied myself that those sufferings here and in Glasgow and other large towns in Scotland are much greater and more general than in towns of equal size in the best regulated parts of Europe, and being thoroughly convinced that the opinions generally entertained in Scotland as to the best means of relieving them are very erroneous, I feel it to be a duty to lay the result of my observations and enquiries before the public. That I do not speak unadvisedly when I use the word *increasing* will appear from the following facts, which, being taken from the records of public institutions, are not liable to the fallacies which might be suspected in the statements of any individual:

"1. The expenditure of the Society for the Relief of the Destitute Sick, the members of which are uniformly men of experience in regard to the habits of the poor, and never grant relief without personal inspection in their own houses of families suffering at once under disease and destitution, has increased from £736, the average of the years 1814-15-16, to £1816, the average of the years 1836-7-8; and the number of individuals receiving relief from them has increased from 3223, the average number in the three former years, to 10,570, the average of the three latter years." (p. 1.)

The increasing number of applicants for accommodation in the Royal Infirmary, and the necessity which was felt a few years ago to institute a house of refuge, by which temporary relief is annually afforded to above 1600 persons, are additional testimonies of the increasing evils referred to.

The causes of the deteriorated condition of the lower classes in the Scottish metropolis are stated to be the diminished proportional expenditure of the higher ranks; the smaller number of wealthy persons who reside permanently in Edinburgh; the reduction in the establishments of the courts of law, and of the different public boards; the failure of building speculations, and the depreciation of property; all of which have necessarily deprived a large number of persons of employment, or rendered their employment irregular and precarious, while

"It is also to be remembered, that this and all other assessed districts in Scotland present a point of attraction to the poor of the numerous districts in Scotland (517 parishes) where there is no assessment for the poor, and often no hospital or other medical charity. The medical charities here are much burdened with cases, often incurable, from those districts; and as long as the relief given in those parts of the country is so small, while the law apportions some allowance (scanty although it be) to all infirm and destitute persons who have lived three years in Edinburgh, I apprehend it will act as a continued bounty on the importation of distressed and half employed families from those districts. Above all, this law has long acted as a bounty on the importation of such families from Ireland; which has gone to such an extremity as fully to justify the observation, 'that if we are to cut off the sources of mendicity we must cut off Ireland.' It need hardly be added, after what has now been stated, that the condition of great numbers of the poor in Edinburgh, particularly during the winter, is one of extreme destitution; approaching in many respects very closely to that which has long been the subject of astonishment and compassion to those who have visited the worst parts of Dublin and other Irish towns." (p. 4.)

To this are annexed extracts from several works descriptive of the unparalleled wretchedness of the lower Irish in their native country, where fever is notoriously in wide-spread and ceaseless operation.

4288

"That there has been a still more rapid increase of destitution, and that similar 'scenes of wholesale human degradation and misery' exist to a still greater extent in Glasgow, is sufficiently shown, *First.* By a few of the facts recorded by the committee appointed there last year to enquire into the cause of the increased assessment: 'The aid to casual poor exhibits more than a threefold increase between 1829 and 1837,' and 'the expenditure for coffins for paupers and their children is nearly *four* times as great now as 1829-30.' *Secondly.* The same is shown by some sentences recording observations made by one of the assistant-commissioners on the handloom enquiry: 'The wynds in Glasgow comprise a fluctuating population of from 15,000 to 30,000 persons. This quarter consists of a labyrinth of lanes, out of which numberless entrances lead into small square courts, each with a dunghill reeking in the centre. Revolting as was the outward appearance of these places, I was little prepared for the filth and destitution within. In some of these lodging-rooms (visited at night) we found a whole lair of human beings littered along the floor, sometimes fifteen and twenty, some clothed and some naked; men, women, and children huddled promiscuously together. Their bed consisted of a layer of musty straw intermixed with rags." (p. 7.)

In respect to Scotland generally, the following account, quoted by Dr. Alison, is stated to be a true picture:

"The female field-labourers, (very numerous here as in every town in Scotland,) when employed, earn only eightpence a day, and are unable to provide anything for the future. Accordingly ceasing to be fit for work about the age of fifty, they inevitably become destitute, and depend for the remainder of their lives on the charity of their neighbours or parochial allowance. The number of such poor women in almost every town in Scotland is distressing to think upon. Though unfit for active exertions, they have a tenacity of life which usually carries them through many years of extreme penury. Habitual piety gives them resignation, sometimes even cheerfulness; but this ought not to blind any enlightened or humane enquirer to the real nature of their situation. The fact is, they live in a condition to which that of most domestic animals is a luxury. The parish rarely offers to such persons more than a shilling a week. Individuals occasionally give them some scraps, but this succour is very trifling. Their mode of life is often altogether a mystery, nothing like the usually understood means of maintaining life being found within their reach." (p. 19.)

Against the lowest depths of destitution there is no security afforded to the wretched poor of Scotland, as there is in the legal provision guaranteed to those of England. It might amuse our English readers (if such a theme were fit for amusement,) to learn the reasons which induce our northern neighbours to keep the parochial relief for the destitute at the lowest rate consistent with the appearance of relief. We have somewhere read an eulogy on the system which gave ample scope for suffering on the part of the poor, on the ground of its conducing to cultivate the feelings and perfect the characters of the rich, by exciting and sustaining their sympathies; and, monstrous as this may appear, it is very nearly what is commonly urged by those who deprecate the introduction of a more effectual system of assessment for the benefit of the destitute. What voluntary contributions have done for the Scottish poor seems to be miserably little, at least in *protecting* them from suffering; and it appears to us a strong argument, independently of its invariable insufficiency, against leaving those who are ever on the verge of destitution, to the tender mercies of a voluntary system, that before the latter can be roused into active operation in the event of sudden reverses in trade or in agriculture, a fearful extent of misery and want has to be endured by the thousands whose heritage is poverty.

"The proportion," says Dr. Alison, "in which the legal relief given in England exceeds that given in Scotland varies considerably. The whole population of 662 unions and parishes in England, to which the new law has been applied, is, in round numbers, 11,166,000, and the sums applied in them to relief of the poor under the new law in 1838 were £4,254,000. The population of Scotland is stated by the committee of the General Assembly at 2,315,000, and the whole sums applied to the relief of the poor, about one half of which are raised by assessment, are £140,496. The whole funds thus applied are nearly six times as great, in proportion to the population, in England as in Scotland. The average expenditure per head on the population in England is stated in the last report of the commissioners to be now reduced to 5s. 10d., and in Wales to be 6s. In Scotland it is less than 1s. 4d." (p. 31.)

In Edinburgh the amount of the legal provision for the poor is 2s. 6d. a head on the population; in Glasgow, Dundee, Paisley, and most of the other Scottish towns it is less than 2s. a head on the population,—less than a third of the expenditure in England. The highest provision granted to a pauper in Edinburgh or in Glasgow (even to a widow with a family) is less than 2s. a week, and the pension to a single male or female pauper is about 1s. a week; whereas in England a widow with four children has from 4s. to 7s. a week, and an aged or disabled man or woman has from 2s. to 4s. Besides, in all Scotland there are but four workhouses, while in England, in May, 1839, there were 587. Scotland appears to equal disadvantage when compared with Hamburg, Holland, many parts of France, and other continental states, in respect to the legal provision for the poor. That this great defect is supplied by the abundance of voluntary contributions is utterly disproved by the amount of misery and want existing in the larger Scottish towns more especially. Dr. Alison also attests the extreme insufficiency of the support given by the public of Edinburgh to those charitable institutions which aim at relieving the prevalent wretchedness. There appears to be no want of support to those institutions in which sickness is to be relieved, but "the funds of several of these institutions, viz. the Infirmary and Dispensaries, are in a great measure supplied by the students of medicine who come from a distance." The funds of other charities, intended for the relief of indigence alone, appear to be miserably defective, and on the decline.

In consequence, as Dr. Alison maintains, of the absence of adequate relief for the poor, disease, and more especially fever, has been on the increase.

"For many years past contagious fever has never been absent from Edinburgh, and there have been three great epidemics of that disease in the last twenty-two years, beginning in 1817, 1826, and 1836, (the last of which has now nearly subsided,) each lasting nearly three years, and each of the last two affecting, I believe, nearly ten thousand persons. The number of fever patients admitted into the Infirmary and Auxiliary Fever Hospital from November 1817 to November 1820, was 3090; from November 1826 to November 1829, it was 4318; and from October 1836 to October 1839, it was 4850." (p. 8.)

It is conceived that not more than half the cases which occurred during these epidemics were removed to the hospitals, and therefore that the whole number affected between 1836 and the close of 1839 must have been at least 10,000. This calculation we believe to be considerably below the actual amount. In Glasgow, it appears from the table contained at p. 10 of Cowan's Vital Statistics of Glasgow, that between 1827 and 1836 not more than one third of the whole cases of fever were received into hospitals; and though the difference of accommodation is in favour of Edinburgh, we doubt if it be so great as the calculation of Dr. Alison, compared with the latter statement, would lead us to infer. That the prodigious amount of fever in the several periods noticed by Dr. Alison was new to Edinburgh is very certain, as appears from a letter from Dr. Gregory to Dr. Clark of Newcastle, dated May 2, 1802; where it is stated that accommodation for fever patients had been opened in the Edinburgh Infirmary twenty-five years previously, and that "the ward for men contains eighteen beds, and that for the women contains fourteen beds, without any crowding; *and have very seldom been full.*"*

"That it is always in persons suffering," says Dr. Alison, after referring to the sufferings of the Irish in 1818, "or who have lately suffered, similar privations and sufferings, and the mental depression and despondency which naturally attend them, that continued fever becomes extensively prevalent, is fully established by the history of all considerable epidemics. The elaborate work of Drs. Cheyne and Barker, shows that this has been strictly true of all the great epidemics which have appeared in Ireland since 1700, each of them lasting fully two years, viz. in 1708, 1720, and 1731, in 1740-41 (after the great frost of 1740), in 1800-1, after the rebellion, the transference of the seat of government to London, and the scarcity of 1799 and 1800; and again, in 1817, after the 'transition from the state of war to that of peace,' and the scarcity of 1816 and 1817. That work contains reports from the most eminent physicians in all parts of Ireland on that great epidemic, all agreeing in the statement, that 'the poor were the greatest sufferers, and the fever seemed to rage among them in a degree proportionate to the privations they had endured.' In Ireland, accordingly, at least during the present century, as the general condition of the poor has been decidedly worse than either in England or Scotland, so contagious fever has never ceased to be more generally prevalent. The same observation applies to the epidemic fever in London after the scarcity of 1800 (the last great epidemic which has occurred there)—to the great continental fever of 1813-14, which followed the track of the French army retreating from Russia, but never made much progress in the victorious allied army—to the epidemic fever of 1817, in Italy, consequent on the scarce year 1816—to the epidemic which affected the British army in Holland after the disastrous retreat from Flanders in 1794—in Portugal after that from Burgos in 1812—and to that which nearly decimated the British legion at Vittoria in 1836." (p. 12.)

If the history of the "petechial typhus," which is a common continental name for the contagious fever in question, were fully detailed it would be found that few wars have ever occurred in Europe without this calamitous attendant. The wars of Maximilian II. in Hungary,—of Francis I. in Italy—of Charles V. and of Charles XII.—of Louis XIV. down to those of our own times, amply justify the title of *war-pest* (kriegspest), as applied to typhus by the Germans. It has not always, indeed, afflicted exclusively armies dejected by defeat, and worn out by the sufferings of a retreat or of a siege; but it must be remembered that these disasters were not necessary in former times to create the most aggravated miseries among the troops. Let any one consult Sir John

* A Collection of Papers, &c., p. 58.—*Newcastle*, 1802.

Smythe's Discourses, and he will find that the very miseries and privations which reign among the modern victims of fever were the common lot of the soldiery in the fifteenth and sixteenth centuries. With a correction of the orthography, the following is an extract to the purpose. "But such covetous men of war, under the pretence (as though their soldiers had been either natural fools or children), did, contrary to all military order, put the greatest part of their soldiers' pay into their own purses, allowing them great scarcity of provisions. By which means it came to pass that divers thousands of their soldiers, partly by hunger and partly by evil lodging, and altogether by the small care and misuse of our such men of war, did perish." (See Chisholm on Contagion.) The French revolution while it drew the horrors of war on Italy and Germany, inundated these countries with the war-pest; the former country being the seat of its ravages in the earlier years of the Republic; and the latter subsequently, more especially in the great epidemics of 1803, in the Austro-Russian campaign of 1806-7, in the Prussian war of 1813, during the deplorable retreat of the French from Russia. It is not only from their history in times of war that other countries afford testimony to the truth of Dr. Alison's views. We have already referred to the fevers of Italy in 1764 and 1817, which occurred in circumstances of great and general privation. Many other epidemics might be mentioned in connexion with the like cause, but we restrict ourselves to the following notice of the events which led to an epidemic at Louviers, in Normandy, in 1770, as detailed by Lepecq de la Cloture. "Let us remember," he says, "that for a number of years the price of provisions had become extremely high; and that suffering having sensibly increased, the commune of Louviers was considerably enfeebled, which obliged the manufacturers to lessen a part of their work; consequently a number of operatives remained without employment, and the wages of others were diminished. What happened then? One did not see, as formerly, the workmen enjoying themselves on fête-days, and drowning a part of their care in wine. No, there was for them no more movements of joy. Sadness was painted in pallid colours on every face. Misery had prostrated their spirit. In short they were reduced for the most part to deprive themselves of some of their wretched furniture, to sell them that they might appease their hunger, and to furnish some morsels of bread to the urgent demands of their children." Four thousand of these workmen were employed in woollen manufactures. Their reverses and sufferings appear to have been identical with those of a similar class among ourselves.

"That the same cause," continues Dr. Alison, "has acted very powerfully in producing the recent epidemics in Scotland appears distinctly from the following considerations. First, it appears from observing the times of these epidemics, the first in Edinburgh beginning in 1817, after two bad harvests, and at the same time as the Irish one; the next in 1826, after the great failures in 1825, and the sudden cessation, particularly of building speculations, in Edinburgh; and the last in 1836, after the great depression of trade both in Glasgow and Dundee, with which towns the lower orders here are much connected, and under the combination of other circumstances already mentioned, which have depressed the condition of the poor in Edinburgh of late years." (p. 13.)

In Glasgow the increase of fever within the same period has been still more remarkable. This is fully illustrated by the first Table in Dr.

Cowan's valuable tract on the Vital Statistics of Glasgow, in which we have the "total number of patients treated in the Glasgow Royal Infirmary from 1795 to 1836, distinguishing the number of fever patients each year." During the first seven years, 484 cases of fever occurred; in the second period of seven years, 512 cases; in the third, 574; in the next, from 1816 inclusive, 3866; from 1823 to 1829 inclusive, 6075; in the last seven, from 1830 to 1836, 11,750. These numbers are not to be considered as representing nearly all the cases of fever which occurred in Glasgow, especially during the latter periods; for between two other hospitals, appropriated to fever alone, 3074 cases were treated during two of the epidemics; and besides there were treated at their own houses between the years 1827 and 1836 inclusive, not less than 6202 cases, by the district surgeons. In the mortality bill for 1839, we find that the total deaths from fever in Glasgow for the five years preceding 31st December, 1839, amounted to 4788, which, at the average mortality of one in every ten cases (which is rather above the actual rate), gives for that period 47,880 cases of fever. In 1837 alone, above 20,000 of these cases are supposed to have occurred: not far short of one in twelve of the whole population. That the same public calamities affected the people of Glasgow which have been noticed as conducing to the prevalence of fever in Edinburgh, cannot be doubted. Yet we observe a passage in Dr. Cowan's work which appears utterly at variance with the proposition of Dr. Alison. Alluding to the increase of fever in Glasgow, he says "this increase, especially during the last seven years, has taken place, not in years of famine and distress, but during a period of unexampled prosperity—a period when the wages of labour have been ample, the prices of provisions comparatively low, and every individual, able and willing to work, secure of steady and remunerating employment." (p. 13.) Without some attention and enquiry this account might be apt to mislead. The first great epidemic in Glasgow accompanied a period, as attested by Dr. Cleland, of great stagnation of trade. He informs us that in 1816-7 so great was the distress of the poor that above 23,000 persons sought aid from the charitable funds; and that this condition did not soon subside appears from another statement of his, that, in 1819, 5256 hand-looms were unemployed. Prior to 1816 there is no record of vast reverses in the trade of Glasgow, nor of the extensive prevalence of fever: since then great fluctuations in the prosperity of that city have been certainly not uncommon, as is signally exemplified by the failures of 1825 and 1835, after each of which years fever underwent a great increase, which continued more or less for several successive years. Dr. Cowan's observation, which we have quoted, referred chiefly to the seven years which preceded 1837; and if the comforts of the people were greater on the whole than they have been since, in like manner the amount of fever among them was much less.

"From the close of 1836," says Dr. Cowan, "one of those periodical depressions in trade, arising from our monetary system, has visited this city, and deprived a large proportion of the population of the means of subsistence. From the existence of secret combinations among the working classes in various departments of trade, but especially among the cotton-spinners, and the 'strikes' which resulted from these combinations, a very large proportion of the inhabitants, in addition to those already suffering from the state of the money market, were suddenly deprived of employment and consequently of the means of procuring food. The high price of coal was the means of diminishing the hours of labour, and consequently the amount of wages in numerous factories, and placed fuel beyond the reach of the lower classes for domestic purposes. And in addition to these sources of misery, the average prices of grain were much higher during 1837 than they had been for some years previously." (p. 34.)

Accordingly the amount of fever, which had been still considerable since the previous epidemics, and had never since its sudden and great increase in 1815-6 fallen nearly so low as it had been before that period of depression, greatly increased, so that according to the calculations of Dr. Cowan it would appear to have risen from 6180 cases in 1835 to 10,092 in 1836, and 21,800 cases in 1837. In the absence of a legal method of relief for the multitudes of able-bodied persons thrown out of employment by the several adverse circumstances which have been detailed, soup-kitchens were opened which daily supplied about 18,500 with food for three months, "but notwithstanding all these exertions, famine and pestilence prevailed to a fearful extent, and the rate of mortality (exclusive of the still-born) rose to 1 in 24·63 of the population." (Cowan, p. 34.)

"Again," observes Dr. Alison, "if these principles as to the connexion of epidemic fever with misery and destitution are correct we may expect to find another illustration of them in Dundee, where there has been, first, a great increase of the population, attracted by the rapid extension of manufactures, then a complete stagnation of trade and suspension of manufactures, throwing great numbers out of employment; and lastly, a prevalent belief among the higher ranks, that 'the older and better system of supporting the poor by voluntary contributions,' ought to be preferred to assessments, and, in consequence, a legal provision, even in the worst times, hardly exceeding, as will afterwards appear, the average of the Scottish country parishes. Accordingly we find that prior to the year 1818, 'little demand was made, comparatively speaking, on the Dundee Infirmary for the reception of fever cases, but during that and the following year the disease raged to such an extent in the town and suburbs, that the house became inadequate to the wants of the community. Since that period the progress of the disease has been various, but on the whole *vastly on the increase.*' The last epidemic appears to have attained its maximum there in 1836, when the fever patients in the infirmary were 773, and the deaths from fever, stated in the mortality bills, (which I understand to be kept very accurately,) are 297. But in the years 1836-7-8-9, fever continued very prevalent, and the deaths from it were 880, the whole number of deaths in these years having been 7160, or 1790 in the year, in a population which, in 1831, was 45,000, but is since supposed to have extended, chiefly, however, by accessions from the country, to above 60,000. From these facts we may infer that nearly 10,000 inhabitants of Dundee, or nearly one in six of the population, must have had fever in these four years. The deaths by it were almost exactly one eighth or 12·5 per cent. of the whole (297 in 1962), and exceeded those by consumption by nearly one third, viz. 297 to 200." (p. 14.)

If the connexion just referred to be really of the nature maintained, we should expect to find that large communities shielded from the excess of wretchedness to which the Scottish and Irish cities are subject, should differ from them in their liability to fever. And the connexion in question will be all the more obvious if the communities which are thus less subject to fever and to destitution, are in other respects in much the same condition as those are where fever and destitution are co-extensive. We find this difference in a very marked degree in our English cities; we find their poor much less exposed to suffering and want; we find them much less subject to fever; but we find them also, as is amply attested by the reports on which we have already commented, and by the recent parliamentary enquiry into the health of the large towns in England, exposed to local nuisances, filth, and effluvia, differing, as far as we can judge from the terms in which these are described, neither in degree nor kind from those to which the poor of the Scottish cities are exposed. In Manchester, with a population of nearly 228,000, it appears that the average annual number of cases of fever treated in the hospital for the seven years ending in 1836 amounted to 497, while in Glasgow, with a population of 244,000, the number treated annually in hospital during the same period amounted to 1842; and that in 1836 the number of cases admitted into the Manchester hospital was 780, and into the Glasgow hospitals 3125. "The prevalence of fever in Glasgow," says Dr. Cowan, "when compared with Manchester, is still more strikingly contrasted by the great change which has taken place in this respect. From 1797 to 1806, both inclusive, the number of the fever patients treated in the Glasgow Infirmary was only 883, while those treated in the Manchester Fever Hospital amounted to 4618." (p. 11.) In Leeds, too, another manufacturing city, with a population at the last census of 123,393, the average annual number of patients treated in hospital during the last seven years, was only 274. In Newcastle and Gateshead, with a population of 57,917, the average number of cases admitted into the institution for the cure and prevention of contagious fever during the like period was only 39 annually. (Cowan, p. 11.) In Carlisle and its vicinity, a population of above 32,000 yielded, on an average of seventeen years before 1838, only 63 cases in the year to the House of Recovery; but in 1838 fever prevailed there epidemically, and affected about 600 persons, of whom 265 were received into the hospital. In Birmingham, where the population is now reckoned at nearly 150,000, the average annual number of cases of fever (including agues) has been for the last seven years only 69. In Sunderland, where the population amounts to nearly 50,000, only seventeen cases were taken into hospital in each year during 1836-7-8. In English towns where extensive manufactories do not exist, the proportion of fever cases is still smaller. In Oxford, for example, a population of 16,000 does not yield to the infirmary five cases of fever in the year.

In London fever has lately prevailed to an unusual extent, especially in 1838; and has affected chiefly those districts which are inhabited by the lowest classes of the population. The total population of these twenty unions is 851,229; which afforded, in 1838, 12,709 cases of fever, or only about one in sixty-seven. If we take the four districts which were the most affected with fever, St. George the Martyr, Bethnal Green, Whitechapel, and Lambeth, we have 6097 cases of continued fever in a population of 253,784, almost exactly that of Glasgow in 1837, where above 20,000 cases of fever occurred in that year of its unusual prevalence. In ordinary years the amount of fever in Glasgow, for nearly ten years past, has been very little short of the number which occurred in these unions during a period signalized as one of an uncommon or epidemic prevalence of fever.

That fever should prevail to a considerable extent in several of the larger cities of England is not to us a matter of surprise. Even although destitution may not exist to *generate* fever, yet no superiority of food and clothing, we are persuaded, can ever protect a community from the extension of that disease by contagion, while the other parts of the United Kingdom are so constantly and extensively afflicted with the malady; for the migratory habits which necessity has imposed on the lowest classes of the population in those parts cannot fail to be the means of introducing the contagion from time to time among their more fortunate neighbours. The proofs of this are to be found, we conceive, in the notorious fact that the lodging-houses, and the dwellings to which the lower Irish have access, are in our towns the places to which fever is most commonly traced or referred. Local peculiarities, also, may give a facility to the diffusion of fever in England, which may detract somewhat, in appearance, from the arguments and views of Dr. Alison. Liverpool, perhaps, affords the most remarkable example having this tendency. That city, in 1836, with a population of 189,242, afforded to the hospital 1700 cases of fever. Considerable as this number is, it will be observed that it does not amount to more than two thirds of the number in Glasgow in 1836, in proportion to the population, and considerably less than half the proportion in Glasgow in 1837. The number which Liverpool would have yielded to the hospital, had its population been equal to that of Glasgow, would be 2272; the number of cases admitted into the Glasgow hospital in 1836 was 3125. The number of fever patients in Liverpool in that year was undoubtedly very great, and from what we have learnt of the comparative plenty which exists among the lower classes there, could not have depended on the destitution of the inhabitants. But there are two circumstances in the case of Liverpool which have caused it at all times to be more affected with fever than any other English town. Forty years ago, when its population did not exceed 63,000, Dr. Currie informs us that the average annual number of cases of fever in Liverpool was about 3000; and that, too, notwithstanding poor-rates that amounted, in 1801, to £28,000. At that time 7000 persons lived in cellars, and 9000 in back houses with very imperfect ventilation. At present it appears from the Parliamentary Report on the Health of Towns, that nearly 40,000 persons reside in the underground cellars, and 86,400 in courts so constructed that ventilation is extremely imperfect; or, as stated in the Report, as if "contrived for the purpose of preventing ventilation." The facilities which are thus afforded for the diffusion of a contagious disease are sufficiently obvious, and are illustrated very emphatically by the late prevalence of smallpox, as well as of fever, in Liverpool. Dr. Duncan, in his evidence, says, "I have facts pointing out the noxious effects of the cellars on the inhabitants with regard to fevers. The proportion of cases of fever occurring among the inhabitants of cellars is about thirty-five per cent. more than it ought to be, calculating the proportion of the inhabitants of the cellars to the whole working population; that shows that the inhabitants of the cellars are much more liable to be affected with fever than the inhabitants of other dwellings." (p. 144.) The other circumstance which renders Liverpool subject to fever is its peculiar exposure to the introduction of contagion by those who frequent the seaport, and by the multitudes of Irish who resort to the city. The fever prevailed to a considerable extent among the seamen of the port; and the number of the Irish in Liverpool are estimated at 60,000. With all these disadvantages, however, fever has not been increasing in Liverpool, as in the Scottish cities. In the beginning of the present century the cases of fever, as we have stated, were annually 3000; in 1840 it is stated that "the average annual number attended during the last five years was upwards of 5000," besides some which, for particular reasons, are not included in this calculation. It would appear, therefore, that while the population of the city has increased from 63,000 to 250,000, the population of the parliamentary borough, that of the parish alone being 190,000, the amount of fever has been only doubled. A material decrease, therefore, has taken place in Liverpool, within the last forty years, in the proportion of fever patients, otherwise the number at present should have amounted to nearly 12,000 annually. This decrease of fever in Liverpool is doubtless to be ascribed both to the increased prosperity of the town and to the check which has been imposed on contagion since 1802, by the establishment of a fever hospital. For in Glasgow, Edinburgh, and Dundee, notwithstanding ample hospital accommodation, contagious fever has been on the increase out of all proportion to the increase of the population. Without such accommodation, we apprehend that the recent history of pestilence in those cities would have rivalled that of the worst European epidemics in the worst times.

We have already referred to the decrease of fever in Manchester since the early part of this century; and it appears from the notice taken of the subject by Dr. Haygarth and Dr. Ferriar, that this decrease is to be ascribed mainly to the influence of the House of Recovery, erected in 1796. That another circumstance should also be taken into account as illustrating the cause of the decline of fever in Manchester, will appear from what Dr. Ferriar says when referring to the causes of the epidemic which prevailed there in 1794. Among these causes were "the want of clothing, and failure even of necessary food, in many families, occasioned by the decay of trade, and the great number of workmen enlisted in the army, who left their children to the slender support which could be earned by the labour of the mother. In many instances I have found that for three or four days before the appearance of typhus in a family, consisting of several children, they had subsisted on little more than cold water. Many of these persons were strangers, and not entitled to, or unable to obtain, the pittance afforded by the poor laws. Even when that relief could be procured, it was very inadequate to the wants of a numerous family. Those who are accustomed to affluence and ease would shudder at the idea of supporting a sickly mother encumbered with the charge of four or five infants on an income of two shillings a week; this, however, is the parochial relief in cases of illness." (Med. Hist., tom. ii.) The diminished proportion of fever patients in Manchester is very strikingly illustrated by the facts, that in the first year of the existence of a fever hospital there were 623 fever patients admitted, and that forty years later, while the population has been increasing so rapidly as to have augmented above 36 per cent. between 1821 and 1831; the number received into hospital in 1836 amounted only to 780.

The decreased mortality in London from fever is very remarkable, as exhibited in the bills of mortality for the last hundred years and upwards.

In 1750 the deaths from fever in London were almost one fifth of the whole mortality; whereas lately, in an epidemic period too, they have not exceeded one twelfth. The annual average of the deaths from fever in London, for the whole of the last century, is above 3000 (including those from scarlet fever), from which we may presume the annual average of fever patients to have been about 30,000; while for the year ending March, 1838, a period of unusual prevalence of fever, we find the worst districts of the city, containing a population of above 850,000, yield only 13,972 cases of synochus, typhus, scarlatina, and intermittent fever: the remainder of the population doubtless yielded a much smaller proportion of cases. In making the comparison between this last period and the past century the difference of population is to be kept in mind. The total number of deaths from fever in 1838, as shown by the returns under the new registration act, was 4078, and from scarlatina 1524, at which time the population may be reckoned at 1,888,800: the mortality from fever was consequently 2·32 in 1000. The mortality from fever has slowly declined since 1838.*

That the improvement in the health of London is in part owing to the introduction of means of separating the sick cannot be doubted; but we must also in part ascribe it to the improved condition of the poor—to the better administration of parochial relief. The complaints on this head made by Willan in 1799, and by Lettsom more recently, leave no room for doubt respecting the favorable change which has since occurred.

The exemption of the large cities in the rest of Europe from epidemics of fever that can bear any comparison with those of Scotland and Ireland, is sufficiently well known; and, according to Dr. Alison, is attested by the great number of foreign medical men who resort to those kingdoms to study the disease with facilities which their own countries do not afford. If poverty can give rise to fever it might be presumed, by those who are ignorant of their condition and circumstances, that the labourers employed about Montfaucon should be subject to it in no ordinary degree, for it may very naturally be supposed that the occupations of that neighbourhood can hold out no attractions except to the destitute. But fever, as we have seen, is not a common disease there, and it is not a little important to know that destitution, contrary to what might have been reasonably conjectured, is not heard of among its population. According to Duchâtelet the people are always employed, to which circumstance he justly ascribes much of their healthiness; and besides, in matter of food, they have been accustomed to a liberal supply of an animal

* The following statement is from Mr. Farr's elaborate article on Vital Statistics, in Maculloch's work, (vol. ii., p. 579,) and may be fully relied on from the well-known accuracy of that able statistician.

MORTALITY IN LONDON.

Deaths to 100,000 living.

| | 1629-35 | 1660-79 | 1728-57 | 1771-80 | 1801-10 | 1831-5 |
|---|---|---|---|---|---|---|
| Fever . . . . | 636 | 785 | 785 | 621 | 264 | 111 |
| Spotted Fever . . | 45 | 90 | ... | ... | ... | ... |
| Plague . . . . | 125 | 1225 | ... | ... | ... | ... |
| Scarlatina . . . | ... | ... | ... | ... | ... | 53 |
| | 806 | 2000 | 785 | 621 | 264 | 164 |

diet—horseflesh—not indeed the most inviting, but apparently wholesome enough.

That Dr. Alison's work may not fail to lessen the evils under which the lower classes of his native country suffer so much must be the wish of every lover of his fellow-men. That the details of misery and suffering which it adduces demand the early and anxious scrutiny of the legislature, appears to be undeniable. We heartily thank him for the zeal with which he has thrown himself into the cause of the poor, and fervently trust that a state of things so deplorable as that which he describes may soon cease to be the reproach of a Christian country. There may be differences of opinion about the way in which so desirable an event may be best achieved, but the necessity of some measure being adopted for the relief and protection of the destitute Scottish poor is placed beyond all doubt. Who cannot sympathize with the feeling of the following eloquent and touching passage?

"If it is the dream of an enthusiast to hope that the law may yet extend a similar protection to such sufferers with us as it does in various other countries, I can only say that it is a dream which will last my lifetime; and that I should wish, by what I have now written, to bequeath it to those who may come after me in the same path of duty, to cheer the hearts of others, as it has done mine, in many a solitary wandering through the abodes of poverty and suffering."

*Note by the Editor.*

It cannot be denied that the cause of fever is involved in much obscurity, and that we still want data on which to rest deductions which will carry with them *unhesitating* conviction. In the actual state of our knowledge it can only be said that the largest number of facts and the best arguments are on one side; but still there are facts and circumstances difficult to be explained on either hypothesis. It may, therefore, be asked, why, entertaining such views, we give a place in our pages to the foregoing article, or at least that part of it relating to malaria, —able, acute, and forcibly argued as all must admit it to be; because, taking as it does the view that fever is not caused by malaria, it might seem to have a tendency to damp the zeal of those who are labouring to improve our towns, by giving the impression that by making sewers, removing filth, opening thoroughfares through densely-populated neighbourhoods, and inducing habits of cleanliness, they were not thereby removing the causes of disease. If this were necessarily the tendency of the arguments of the contagionists, it would not only, in the present state of our information, be culpable to support them, but on this very account should their validity be suspected; but in a practical point of view both parties are in accordance. The one says, that by cleanliness and ventilation you remove at once the cause of the disease: the other, that you remove the causes which propagate the disease when once it is in existence; for that whatever tends to concentrate the poison, or to weaken in any degree the vital powers, renders the victims more numerous. The latter view of the subject is, therefore, if anything, perhaps the one which is more persuasive to an extended benevolence. Those who regard fever as dependent on malaria, would be more likely to pursue but one object; the cleansing of the poisonous atmosphere of filthy, crowded rooms and streets: the contagionist would go farther and say this is not enough, for unless you relieve that debilitated state of body and mind which arises from meager poverty, you leave unremedied one of the most powerful causes of the propagation of the malady.

